# Supplementary material for: Hepatitis B virus prevalence and vaccine antibody titers in children HIV exposed but uninfected in Botswana
Source: PLoS One. 2020 Aug 7;15(8):e0237252. doi: 10.1371/journal.pone.0237252 (PMC7413399; doi:10.1371/journal.pone.0237252)
Supplement: Attachment — Submitted filename: Response to reviewers_Final.docx [file pone.0237252.s001.docx]

Botswana Harvard AIDS Institute Partnership

Private Bag BO320

Gaborone, Botswana

19^th^ May, 2020

Editorial Team

PLOS ONE

Dear Editors

**RE: PLOS ONE [PONE-D-20-06802] - [EMID:2822b464639a2f2b] point-by-point response letter**

We greatly appreciate all the comments from the reviewers, and we believe they have led to the improvement of the manuscript. Here we present our responses to the reviewer comments. The responses are in red.

Please do not hesitate to contact us for any further comments.

Sincerely,

Simani Gaseitsiwe, PhD

Laboratory Director at Botswana Harvard AIDS Institute Partnership (BHP)

& Research Associate at Harvard T.H. Chan School of Public Health (HSPH)

Phone: L +267 3902671 ext 2216

M +267 7171 7317

Fax: + 267 390 1284

Address of additional requirements:

1. Please ensure that your manuscript meets PLOS ONE's style requirements, including those for file naming. The PLOS ONE style templates can be found at

<http://www.journals.plos.org/plosone/s/file?id=wjVg/PLOSOne_formatting_sample_main_body.pdf> and <http://www.journals.plos.org/plosone/s/file?id=ba62/PLOSOne_formatting_sample_title_authors_affiliations.pdf>

The manuscript was formatted to meet PLOS ONE's style requirements

Lines 130, 148 and 311: The tittle was corrected to sentence case.

Line 23: Corresponding author email was moved from line 22 to line 23 and the initials were moved next to the email address in parentheses.

Lines 198, 223, 238 and 283: The letter f in fig was capitalized

Tables 1-3 were corrected to cell-based in Microsoft Word

2. Please provide additional details regarding participant consent. In the ethics statement in the Methods and online submission information, please ensure that you have specified what type of consent you obtained (for instance, written or verbal, and if verbal, how it was documented and witnessed).

The word “written” has been included in line 101 of the methods section under study participants. The sentence now reads: Mothers provided written informed consent for participation in the Mpepu study, and for storage of residual samples.

3. In your Data Availability statement, you have not specified where the minimal data set underlying the results described in your manuscript can be found. PLOS defines a study's minimal data set as the underlying data used to reach the conclusions drawn in the manuscript and any additional data required to replicate the reported study findings in their entirety. All PLOS journals require that the minimal data set be made fully available. For more information about our data policy, please see <http://journals.plos.org/plosone/s/data-availability>.

**Data Availability Statement:** The subset of data used for these analyses has been cleaned and extracted from the larger, complete database of the ‘Mpepu study’. This dataset is ready for sharing with interested researchers after going through the appropriate, and mandatory, regulatory procedures in accordance with Botswana National guidelines. Requests for data can be made by applying to the Botswana Ministry of Health and Wellness Human Research Development Committee (HRDC). IRB contact: Seeletso

Reviewer #1: Summary

This is a descriptive study reporting HBV specific antibody levels in 18-month-old HIV-exposed uninfected (HEU) infants in the Botswana setting. The manuscript shows that early immunization of infants induces antibody levels above the protective threshold in >90% of vaccine recipients, and in this cohort, no HBV infection were detected prompting good vaccine responses. The paper is well written, concise and scientifically sound and adds on existing literature regarding vaccine responses in HEU infants and thus is appropriate for this journal. Additional statistical analysis could be included to determine other factors that may be associated with infant antibody levels.

Major Comments

Introduction:
1. Line 69: strike out “exposure”

“Exposure” in line 69 was removed. The sentence now reads: HIV-exposed but uninfected (HEU) children are exposed to HIV in utero or via breast milk but remain HIV-uninfected; however, there are limited data on the risk of HBV transmission via breastfeeding

Methods:

1. Description of infant demographics in the study need to be included in methods or the result section.

Table 1 under the results section has been included. The table contains infant demographics among others sex, birth weight, administration of the birth dose and mode of feeding for the infants. The Mean birth weight was also added in line 177-178.

2. Authors should describe method for classification of infants as HEU even if mentioned in the parent study.

Classification of children as HEU has been added in line 109. The sentence reads: HEU children were defined as HIV negative children who were born to HIV positive mothers.

3. Line 90: mentions sample collection at 14-28 days after birth but HB titres were not measured at this time point – need additional information why this was not done.

The reason why anti-HBs titers were not measured at this point was because we wanted to measure the titers after the infants had received all the HBV vaccine doses. In Botswana infants are vaccinated at birth, 2, and 4 months. However, we decided to test 18 months old samples to also avoid quantifying passive maternal antibodies and classifying them as vaccine antibody titers. The statement “The 18 months visit was chosen in order to measure anti-HBS titres after completion of HBV vaccine doses and to avoid quantifying passive maternal anti-HBS” was added in lines 107-108.

4. Also, was there a reason for not measuring HB titres in mothers?

Infant vaccination in Botswana started in 2000 which is 20 years ago. However, the mean age of the mothers stands at 31.8 years which means majority of them are unlikely to have received the HBV vaccine hence the decision not to test for anti-HBs titers in the maternal samples.

5. Re-phrase line 130-133, difficult to follow.
Lines 130-133, now line 141-146 were rephrased and now read: The PCR products were purified using the QIAquick® PCR purification kit (QIAGEN, Mannheim, Germany) according to manufacturer’s specification with an elution volume of 30µL. Sequencing PCR was done using primers HBV 381 and HBV 801 and cleaned up using ZR DNA Sequencing Clean-up Kit™ (Zymo, Irvine, CA, USA).

6. Consider rephrasing line 140.
Line 140, now line 152 was rephrased to: Genotypes and resistance mutations were determined using the Stanford database

7. Line 154 “the whole surface region…surface open reading frame (ORF) was used… Do you mean surface region of HBsAg
“Whole surface region” and “surface open reading frame” were used to refer to the HBV surface region. They were rephrased to HBV surface region. The sentences now in line 166-169 now read: A tree for sub-genotype A1 and genotype E sequences from this study and the respective GenBank references for the HBV surface region were constructed. The HBV surface region was used to determine the clustering of HBV strains from this study relative to other Botswana HBV sequences and few sequences from the rest of the world.

Results:
1. No demographic description included for the children – give detail of median age at time of plasma sampling, did all infants receive all doses of the vaccine?

The study only included samples collected at 18 months, which has been highlighted in line 105-107. Unfortunately, information about whether the infants received all doses was not available, we only had information about administration of the HBV birth dose which has been included in table 1

2. Line 164 - need a breakdown of when the infants received the vaccine – would this had correlated with antibody levels?

Data about when the infants received doses given at 2, 3 and 4 months of life were not available from the parent study (Mpepu study). Only HBV birth dose information was available, provided in table 1. No correlation was found between timely administration of the HBV birth dose and antibody levels. Line 190-193 explains this: For the HEU children with anti-HBs titre of less than 10mIU/mL, 2 of 3 HEU children had received a timely administration of the HBV birth dose, while 1 did not receive the HBV birth dose within the first 24 hours of birth.

3. Consider re-phrasing line 165-168 for clarity.

Line 165-168, now line 186-190 has been rephrased and now reads: Assessment of vaccine antibody response in HEU children, using ≥10mIU/mL as the threshold for protective immunity, showed that 3 (1.1%) of 269 (95% CI, 0.2%, 3. 2%) HEU children had an inadequate immune response (<10 mIU/mL), while 266 (98.9%) of 269 (95% CI, 96.8%, 99.8%) had protective immunity.

4. Line 171 – The use of the word “about” is confusing, was 170 not the exact figure?

170 was the exact figure. The word “about” in line 194, previously line 171 has been removed. The sentence now reads: However, for the ≥100 mIU/mL threshold, 170 (63.2%) of 269 HEU children had anti-HBs titres of greater than 100 mIU/mL showing complete protection, 96 (35.7%) of 269 HEU children had anti-HBs titers ranging between 10-100 mIU/mL showing partial immunity whilst 3 (1.1%) of 269 HEU children had anti-HBs titers of <10mIU/mL classified as no protection

5. Was the range of antibody levels associated with timing of vaccination or collection of plasma samples? Are there any other maternal or infant factors associated with antibody levels e.g sex, maternal HIV viral load or CD4 levels?

The range of antibody levels was not associated with the timing of vaccination as explained in lines 190-193: For the HEU children with anti-HBs titre of less than 10mIU/mL, 2 of 3 HEU children had received a timely administration of the HBV birth dose, while 1 did not receive the HBV birth dose within the first 24 hours of birth.

The range of antibody levels could be associated with gender because all the 3 HEU children with anti-HBs titres of <10mIU/mL were male. A sentence has now been added in line 193 which reads: All the HEU children with anti-HBs titers of less than 10mIU/mL were male.

6. No mention of antibody level testing in maternal samples.
Infant vaccination in Botswana started in 2000 which is 20 years ago. However, the median age of the mothers stands at 31.8 years (table 2) which means majority of them are unlikely to have received the HBV vaccine so we decided not to test for anti-HBs titers in the maternal samples.

Discussion:
1. Line 221: difficult to determine protection in the absence of exposure.

Line 221 refers to protection as per the widely used and acceptable threshold of ≥10mIU/mL which is thought to be enough to prevent HBV transmission. The children in this study were exposed to HBV both through vertical and horizontal transmission. Vertical transmission in the sense that 5 mothers tested positive for HBsAg but did not infect their children due to their positive immune response to the vaccine. Data on the mode of feeding show that 1 HBsAg positive mother had been breastfeeding her infant, but the infant tested negative for HBsAg.

2. Line 226: missing the word “vaccine”

The word vaccine has been added to where appropriate in line 251, previously line 226. The sentence now reads: .A recent WHO report found that in Botswana, 94% of new-born infants complete the 3-dose schedule of HBV vaccination, while the timely hepatitis B birth dose vaccine coverage was 74%

3. Line 228-230: missing reference

Line 251-253, previously line 228-230, was referring to demographics of our study samples which has now been included in table 1, not from the parent Mpepu study. The line has been rephrased and now reads: Timely administration of the hepatitis B birth dose for our study in 304 HEU children was 75% which is similar to the WHO estimates (table 1).

4. Line 240: consider rephrasing to – “.not receiving the HBV primary vaccine dose at birth”

Line 261, previously line 240, has been rephrased to include “not receiving the HBV primary vaccine dose at birth”. The sentence now reads: In Malawi, a retrospective longitudinal study was conducted to assess the immune response to HBV vaccine in HIV-exposed children of unknown HIV status, who had been vaccinated at 6, 10, and 14 weeks of age and to investigate the correlation between not receiving the HBV primary vaccine dose at birth and HBV infection acquisition

5. Line 243 Should be in the results section.

Line 267, previously line 243 has been moved from the discussion and now forms part of the results (line 202-204).

Minor issues
1. “However” should be used to contrast preceding sentences or phrases and it’s use in Line 29, 66 and 81 is a bit misleading.

The word “however” has been deleted from the text. Line 30 previously line 29 reads: To the best of our knowledge, since the introduction of HBV vaccination, there have been limited data for vaccine response to HBV and its impact on early childhood HBV infections among HIV exposed uninfected (HEU) children in Botswana. Line 66 reads: This figure was reduced to 2.9% among children who received the immunoprophylaxis, thereby highlighting the significant benefit of immunization. Line 87, previously line 81 reads: To the best of our knowledge, data on HBV vaccine responses in HEU children in Botswana remain unknown.

2. Double check for punctuation following in text reference inserts.

Double checks for correct punctuation have been done and line 122-125 have been corrected.

3. Line 172 – preference of the use “greater than” instead of “more than”

“More than” has been removed from the sentence in line 195, previously line 172 and “greater than” has been added. The sentence now reads: However, for the ≥100 mIU/mL threshold, 170 (63.2%) of 269 HEU children had anti-HBs titres of greater than 100 mIU/mL showing complete protection, 96 (35.7%) of 269 HEU children had anti-HBs titers ranging between 10-100 mIU/mL showing partial immunity whilst 3 (1.1%) of 269 HEU children had anti-HBs titers of <10mIU/mL classified as no protection

Reviewer #2:

Intro:

Line 78: Will be helpful to mention the dosing (i.e. 6, 10 and 14week) of HBV vaccine
Also, might be important to mention the Botswana guideline as respect breast feeding among HIV infected mothers
Information on the dosing of the vaccine has been added. The sentence in line 76-79 now reads: Botswana adopted the World Health Organisation (WHO) recommendation in 2000 to administer a birth dose within 24 hours of birth followed by three additional doses given at 2, 3 and 4 months of life to prevent perinatal and early horizontal HBV transmission.

Data about Botswana infant feeding guidelines with respect to HIV positive mothers that were in place at the time of sampling (2011-2013) have been added in line 82-86. The sentences now read: Infant feeding guidelines by Botswana Ministry of Health and Wellness (MoHW) in 2011 recommended that HIV positive women use formula feeding for the ﬁrst 6 months of life only when it was acceptable, feasible, affordable, sustainable and safe (AFASS). In instances where formula feeding was not AFASS, HIV positive mothers were recommended to breastfeed their infants.

Method:
not clear how the samples were stored (i.e. storage condition of the samples). Information might be helpful to readers.

Information about storage conditions of the samples have been added. The sentence in line 93-95 now reads: Residual plasma samples from the Mpepu study were stored at -80$℃$ in an ultra-low freezer after completion of the study.

Result:
According to your analysis in line 162, two hundred and twenty nine (75%) had timely vaccination while 75 didn’t (that give a total of 304 HEU). However, in line 165 to 168, you seem to analyse the data for 269 of the 304 HEU. So what about the other 35 HEU? It may be better to have a table to visual these key results rather than have them only in text.
During the study we encountered a problem of low volume samples for 35 of the infant samples. As a result, we did not have the required 75µl to measure anti-HBs titers in those samples. A sentence has been added in line 183-184 which reads: The residual volume of plasma in 35 (11.5%) of 304 HEU children samples was inadequate to measure anti-HBs titers, so only 269 samples were available for testing

Why median age of mothers and not mean? Were the data not normally distributed? Could you explain this?

Normal distribution has been checked in all the maternal demographics using the density histograms. Results show that only age and hgb data were normally distributed hence a change was made, in table 2, to use the mean instead of the median. Other parameters did not show normal distribution so median was used instead of the mean.

For infants, the density histogram did not show normal distribution in anti-HBs titres so the median was used. For the birth weight, the graph shows normal distribution so the mean was used instead of the median. A sentence has been added in line 177-178 which reads: The Mean birth weight was 2.92 kg (95% CI, 2.87- 2.97).

Despite being a retrospective study, it will be great to have some demographic information about the children: birth weight, feeding pattern (i.e. breast fed or formula fed) etc. Or at the least, reference the original published work that reported these information if available.

Table 1 under the results has been included. The table contains infant demographics among others sex, birth weight, administration of the birth dose and mode of feeding for the HEU infants.
