# Supplementary material for: Hepatitis B virus prevalence and vaccine antibody titers in children HIV exposed but uninfected in Botswana
Source: PLoS One. 2020 Aug 7;15(8):e0237252. doi: 10.1371/journal.pone.0237252 (PMC7413399; doi:10.1371/journal.pone.0237252)
Supplement: Attachment — Submitted filename: response to reviewers PONE-D-20-06802R2.doc [file pone.0237252.s002.doc]

The following changes have been made:

**Title**

Line 1-2: “HIV exposed” has been removed from the title and “children HIV exposed but uninfected” has been added. The title now reads “Hepatitis B virus prevalence and vaccine antibody titers in children HIV exposed but uninfected in Botswana”

Line 4-5: HIV positive has been removed from the short title and “children HIV exposed but uninfected” has been added. The short title now reads “HBV vaccine antibody titers in children HIV exposed but uninfected”

**Abstract**

Line 32: “HIV exposed but uninfected” has been removed from the sentence and “children HIV exposed but uninfected” has been added. The sentence now reads “To the best of our knowledge, since the introduction of HBV vaccination, there have been limited data for vaccine response to HBV and its impact on early childhood HBV infections among children HIV exposed but uninfected in Botswana.”

Line 35-36: “HIV exposed but uninfected” has been removed from the sentence and “children exposed to HIV” has been added. The sentence now reads “To determine the prevalence of hepatitis B surface antigen (HBsAg) and HBV vaccine response in 18 months old children HIV exposed but uninfected in Botswana.”

Line 37: “HEU” has been removed from the sentence. The sentence now reads “Stored plasma samples from 304 children at 18 months of age and 287 mothers from delivery were tested for HBsAg.”

Line 40-41: “HEU children” has been removed from the sentence and “children exposed to HIV” has been added. The sentence now reads, “Plasma samples from children exposed to HIV were tested for hepatitis B surface antibody (anti-HBs) titers.”

Line 42: “HEU” has been removed from the sentence. The sentence now reads, “No children (0 of 304) were positive for HBsAg at 18 months while 5 (1.74%) of 287 HIV-positive mothers were HBsAg positive.”

Line 46: “HEU” has been removed from the sentence. The sentence now reads, “Three (1.1%) of 269 children had an inadequate vaccine response (<10 mIU/mL), while 266 (98.9%) of 269 had protective immunity.”

Line 48: “HEU” has been removed from the sentence. The sentence now reads, “However, when using the ≥100mIU/mL threshold, only 170 (63.2%) of 269 children had complete protection.”

Line 49-50: “HEU children” has been removed from the sentence and “children HIV exposed but uninfected” has been added. “Were” has been changed to “was”. The sentence now reads,” No HBsAg positivity was identified in a cohort of children HIV exposed but uninfected.”

**Introduction**

Line 69-70: “HEU children” has been removed from the sentence. The sentence now reads, “Some children exposed to HIV in utero or via breast milk may remain HIV-uninfected; however, there are limited data on the risk of HBV transmission via breastfeeding.”

Line 71-73: “HEU” has been removed from the sentence and “exposed to HIV” has been added. “HIV negative” has been removed and replaced by “mothers without HIV”. The sentence now reads, “HBV vaccine response among children exposed to HIV has been reported to be less robust compared to children born to mothers without HIV.

Line 73-74: “HEU” has been removed from the sentence and “exposed to HIV” has been added. The sentence now reads, “This places children exposed to HIV at a higher risk of HBV transmission in the presence of high viral DNA levels in HIV/HBV co-infected mothers.”

Line 84: “HIV positive women” has been removed from the sentence and replaced with “women living with HIV”. The sentence now reads, “Infant feeding guidelines by Botswana Ministry of Health and Wellness (MoHW) in 2011 recommended that women living with HIV use formula feeding for the ﬁrst 6 months of life only when it was acceptable, feasible, affordable, sustainable and safe (AFASS).”

Line 86-87: “HIV positive” has been removed from the sentence and replaced with “mothers living with HIV”. The sentence now reads, “In instances where formula feeding was not AFASS, mothers living with HIV were recommended to breastfeed their infants.”

Line 88-89: “HIV exposed but uninfected” has been removed from the sentence and “children HIV exposed but uninfected” has been added. The sentence now reads “To the best of our knowledge, data on HBV vaccine responses in children HIV exposed but uninfected in Botswana remain unknown.”

Line 90-91: “HEU” has been removed from the sentence and “children HIV exposed but uninfected” has been added. The sentence now reads, “We sought to determine the prevalence of HBsAg positivity and vaccine response in 18 months old children HIV exposed but uninfected in Botswana.”

**Methods**

Line 99: “HEU” has been removed from the sentence and “exposed to HIV” has been added. The sentence now reads, “The objective of the Mpepu study was to assess the efficacy and safety of infant cotrimoxazole versus placebo prophylaxis taken from 14-18 days through 15 months of life in children exposed to HIV”

Line 106-107: “HEU” has been removed from the sentence and “children HIV exposed but uninfected” has been added. The sentence now reads, “Archived plasma samples from children HIV exposed but uninfected and their mothers collected at 18 months and at delivery respectively were used for this study.”

Line 110-112: “HEU” has been removed from the sentence and “children HIV exposed but uninfected” has been added. “HIV negative” has been removed from the sentence and replaced with “children without HIV”. “HIV positive” has also been removed from the sentence and replaced with “mothers living with HIV”. The sentence now reads, “Children HIV exposed but uninfected were defined as children without HIV who were born to mothers living with HIV.”

**Results**

Line 173: “HEU” has been removed from the sentence and “children HIV exposed but uninfected” has been added. The sentence now reads, “Samples from 304 children HIV exposed but uninfected were tested (154 females and 150 males).”

Line 175-177: “HEU” has been removed from the sentence. The sentence now reads, “Two hundred and twenty-nine (75%) of the 304 children had received a timely (within the first 24 hours) administration of the HBV birth vaccine, while 75 (25%) of 304 children had not received the vaccine timely (Table1).”

Line 179: “HEU infant” has been removed from table 1 title and “for children exposed to HIV” has been added. The title for table 1 now reads, “Demographics for children exposed to HIV (n=304)”

Line 183: “HEU” has been removed from the sentence. The sentence now reads, “The residual volume of plasma in 35 (11.5%) of 304 children samples was inadequate to measure anti-HBs titers, so only 269 samples were available for testing.”

Line 185: “HEU” has been removed from the sentence. The sentence now reads, “The median anti-HBs titer in 269 children was 174 mIU/mL [IQR: 70, 457]”

Line 186-189: “HEU” has been removed from the sentence. The sentence now reads, “Assessment of vaccine antibody response in children, using ≥10mIU/mL as the threshold for protective immunity, showed that 3 (1.1%) of 269 (95% CI, 0.2%, 3.2%) children had an inadequate immune response (<10 mIU/mL), while 266 (98.9%) of 269 (95% CI, 96.8%, 99.8%) had protective immunity”

Line 189-192: “HEU” has been removed from the sentence. The sentence now reads, “For children with anti-HBs titre of less than 10mIU/mL, 2 of 3 children had received a timely administration of the HBV birth dose, while 1 did not receive the HBV birth dose within the first 24 hours of birth”

Line 191-192: “HEU” has been removed from the sentence. The sentence now reads, “All children with anti-HBs titres of less than 10mIU/mL were male.”

Line 192-197: “HEU” has been removed from the sentence. The sentence now reads, “. However, for the ≥100 mIU/mL threshold, 170 (63.2%) of 269 children had anti-HBs titres of greater than 100 mIU/mL showing complete protection, 96 (35.7%) of 269 children had anti-HBs titers ranging between 10-100 mIU/mL showing partial immunity whilst 3 (1.1%) of 269 children had anti-HBs titers of <10mIU/mL classified as no protection (Fig 1).”

Line 199: “HEU” has been removed from the figure 1 title. The title now reads, “Fig 1. Anti HBs titer ranges for children plasma samples”

Line 202: “HIV positive” has been removed from the sentence and replaced with “mothers living with HIV”. The sentence now reads, “We report a 1.74% (5/287) (95% CI, 0.6- 4.0) prevalence of HBsAg in a cohort of mothers living with HIV with a mean age of 31.8 years (95% CI, 27.1- 32.5) at delivery (Table 2).”

Line 209: “HIV positive” has been removed from the title of table 2 and replaced with “mothers living with HIV”. The title now reads, “Table 2. Demographics of mothers living with HIV"

Line 211: “HIV positive” has been removed from the sentence and replaced with “mothers living with HIV”. The sentence now reads, “All 287 mothers living with HIV received antiretroviral treatment (ART).”

**Discussion**

Line 227-228: “HEU” has been removed from the sentence and “children HIV exposed but uninfected” has been added. The sentence now reads, “This is the first study investigating HBsAg prevalence among children HIV exposed but uninfected in Botswana.”

Line 228-230: “of HEU children” and “at 18 months” have been removed from the sentence. The sentence now reads, “We report the absence of HBsAg positivity (0%) and relatively strong HBV vaccine responses in this cohort by 18 months of age.

Line 231: “are” has been removed and replaced with “were” to correct the tense. The sentence now reads, “The results in this cohort reflect public health targets promoted by the WHO African Regional Committee in 2014 where countries were challenged to reduce chronic HBV infection incidence to < 2% in children less than 5 years of age by 2020.”

Line 234-236: Line 110-111: “HEU” has been removed from the sentence and “exposed to HIV” has been added. “HIV positive” has also been removed from the sentence and replaced with “mothers living with HIV”. The sentence now reads, “The absence of HBsAg positivity in the children exposed to HIV cohort may be attributed to the low HBV prevalence in their mothers living with HIV as well as high HBV vaccine antibody titers in the children (Fig 1)”

Line 236-240: “HEU” has been removed from the sentence. The sentence now reads, “For the ≥100 mIU/mL threshold, 170 (63.2%) of 269 children had anti-HBs titres of greater than 100 mIU/mL showing complete protection, 96 (35.7%) of 269 children had anti-HBs titers ranging between 10-100 mIU/mL showing partial immunity whilst 3 (1.1%) of 269 children had anti-HBs titers of <10mIU/mL classified as no protection.”

Line 241: “HEU infants” has been removed from the sentence and replaced with “children”. The sentence now reads, “All three children with anti-HBs titers of <10mIU/mL were male which could suggest that sex‑based immunological differences affected the dynamics of the immune response.”

Line 244: “HEU” has been removed from the sentence and replaced with “the”. The sentence now reads, “For the ≥10mIU/mL threshold, the majority of the children (98.9%) developed protective immunity, implying that the HBV vaccine given in infancy in Botswana is highly protective against chronic HBV infection.”

Line 246: “the HEU” has been removed from the sentence. The sentence now reads, “However, children with anti-HBs titres of less than 100mIU/mL may require an additional booster dose to achieve adequate immunity.”

Line 251: “HEU” has been removed from the sentence and “exposed to HIV” has been added. The sentence now reads, “Timely administration of the hepatitis B birth dose for our study in 304 children exposed to HIV was 75% which is similar to the WHO estimates (Table 1).”

Line 258-259: “HEU” has been removed from the sentence and “exposed to HIV” has been added. “Of unknown HIV status” has also been removed from the sentence, The sentence now reads, “In Malawi, a retrospective longitudinal study was conducted to assess immune response to HBV vaccine in children exposed to HIV, who had been vaccinated at 6, 10, and 14 weeks of age and to investigate the correlation between not receiving the HBV primary vaccine dose at birth and HBV infection acquisition.”

Line 266-268: “HIV positive” has been removed from the sentence and replaced with “mothers living with HIV”. The sentence now reads, “The 1.74% HBsAg prevalence in mothers living with HIV was lower than the reported prevalence from previous studies in pregnant women living with HIV which was reported as 3.1-3.8% in Botswana”

Line 271: “HIV positive” has been removed from the sentence and replaced with “mothers living with HIV”. The sentence now reads, “Among the 5 mothers living with HIV who tested positive for HBsAg, none of their children were HBsAg positive.”

Line 278-279: “from mothers living with HIV” and “HBV” have been added to the sentence which now reads, “Genotyping results showed that 4 of 5 samples from mothers living with HIV had HBV genotype A1 which is associated with high risk of horizontal transmission, and 1 sample had genotype E”

Line 282-283: “HEU” has been removed from the sentence and “exposed to HIV” has been added. The sentence now reads, “First, few children exposed to HIV were at risk for vertical HBV infection, as the HBsAg prevalence among their mothers as well as their HBV DNA levels were low.”

Line 291: “HEU” has been removed from the sentence. The sentence now reads, “Third, the HBV vaccination records of the children showed administration of the birth dose, there were no data on whether they completed all their doses which might have an effect on the HBV vaccine antibody titers.”

**Conclusion**

Line 296-298: “HEU” has been removed from the sentence and “exposed to HIV” has been added. “HIV positive” has also been removed from the sentence and replaced with “mothers living with HIV”. The sentence now reads, “We report low HBV prevalence in mothers living with HIV and no cases of HBsAg positivity in 18 month-old children exposed to HIV in Botswana.”

Line 298-299: “the HEU” and “in this cohort” have been removed while “exposed to HIV” has been added. The sentence now reads, “Reassuringly, there was a high response to the HBV vaccine among children exposed to HIV in Botswana.”

Line 302: “HEU” has been removed from the sentence and “exposed to HIV” has been added. The sentence now reads, “Although a very small number of children failed to achieve protective levels of vaccine titers and might be at risk of HBV infection, these results demonstrate low risk and good vaccine protection among children exposed to HIV in Botswana, meeting the WHO-established targets for < 2% paediatric HBV prevalence in the region.”

Line 304: “HEU” has been removed from the sentence. The sentence now reads,” However, 35.7% of the children did not achieve complete protection so further studies on this group are warranted.”
2. Additionally, Table 1 needs some editing. Also there is no need to specify both % male AND percent female unless there were some infants with unassigned sex at birth (i.e. that the % does not add up to 100).
Table 1 has been edited. Information about female percentages has been removed from table 1 because there were no infants with unassigned sex.

3. Finally, line 177: "Mean" should not be capitalised as its in the middle of a sentence, and new-born should not be hyphenated (line 247).

“Mean” in line 177 has been changed to “mean”. This sentence now reads “The mean birth weight was 2.92 kg (95% CI, 2.87- 2.97).”

Hyphenation has been removed from the word “new-born” in line 248. The sentence now reads, “A recent WHO report found that in Botswana, 94% of newborn infants complete the 3-dose schedule of HBV vaccination, while the timely hepatitis B birth dose vaccine coverage was 74%.”

Yours sincerely,

Simani Gaseitsiwe PhD

Principal Investigator at Botswana Harvard AIDS Institute Partnership (BHP)

& Research Associate at Harvard T.H. Chan School of Public Health (HSPH)

Tel: +267 3902671/ +267 71717317

Email: [sgaseitsiwe@bhp.org.bw](mailto:sgaseitsiwe@bhp.org.bw)
